# Hepatitis C virus RNA viremia in Central Africa.

**DOI:** 10.3201/eid0503.990330

**Published:** 1999

**Authors:** N Cancré, G Grésenguet, F X Mbopi-Kéou, A Kozemaka, A Si Mohamed, M Matta, J J Fournel, L Bélec


of age. Sex was not significantly associated (mean male to female sex ratio was 32.4:18 for *S. typhi* and 15.8:10.6 for *S. paratyphi A*).

*S. typhi* has become increasingly sensitive to amoxycillin, chloramphenicol, and gentamicin, increasing from 75.1% in 1994 to 96.6% in 1998 for amoxycillin, from 71.9% in 1994 to 91.6% in 1998 for chloramphenicol, and from 96.4% to 100% for gentamicin. *S. paratyphi A* strains have remained uniformly sensitive (100%) to all antibiotics (amoxycillin, chloramphenicol, and gentamicin, as well as ciprofloxacin and ceftriaxone) used in the treatment of enteric fever. In light of reports of multidrug resistance in *S. typhi,* especially to quinolones, continued surveillance and monitoring of antimicrobial sensitivity of *S. paratyphi A* strains are needed.

The increase in proportion of *S. paratyphi* A cases, which may be due to a high degree of clinical suspicion (with mild fever cases investigated for enteric fever), changing host susceptibility, or even change in the virulence of the organism, should be further investigated.

**Seema Sood, Arti Kapil, Nihar Dash, Bimal K. Das, Vikas Goel, and Pradeep Seth**
All India Institute of Medical Sciences,
New Delhi, India

### References

1. Richens J. Typhoid and paratyphoid fevers. In: Oxford textbook of medicine. Weatherall DJ, Ledingham JGG, Warrell DA, editors. Vol 1. 3rd ed. London:: Oxford Medical Publication; 1996. p. 560-8.
2. Saxena SN, Sen R. *Salmonella paratyphi A* infection in India: incidence and phage types. Trans Royal Soc Trop Med Hyg 1966;603:409-11.
3. Kumar R, Sazawal S, Sinha A, Sood S, Bhan MK. Typhoid fever: contemporary issues as related to the disease in India. Round Table Conference Series on Water Borne Diseases. 12th ed. Ranbaxy Science Foundation, New Delhi, 1997;2:31-6.
4. Kapil A, Sood S, Reddaiah VP, Das BK, Seth P. Partyphoid fever due to *Salmonella enterica* serotype paratyphi A. Emerg Infect Dis 1997;3:407.
5. Thong K, Nair S, Chaudhry R, Seth P, Kapil A, Kumar D, et al. Molecular analysis of *Salmonella paratyphi A* from an outbreak in New Delhi, India. Emerg Infect Dis 1998;4:507-8.
6. Collee JG, Duguid JP, Fraser AG, Marmion BP. Mackie and Mc Cartney practical medical microbiology: laboratory strategy in the diagnosis of infective syndromes. 13th ed. London (UK): Churchill Livingstone; 1989. 601-7.
7. Stokes EJ, Ridgway GL. Clinical bacteriology: anti-bacterial drugs. 5th ed. London: Edward Arnold; 1980. p. 205-19.

### Hepatitis C Virus RNA Viremia in Central Africa

**To the Editor:** Epidemiologic serosurveys have demonstrated high prevalence (6%-15%) of hepatitis C virus (HCV) infection in adults in sub-Saharan Africa (1-4). Although possible false-positive HCV serologic test results have been reported in Africa, HCV prevalence rates suggest a high rate of chronic infection among persons with anti-HCV antibodies (5,6). We have focused on HCV RNA infectivity of blood from donors attending the National Blood Center in Bangui, Central African Republic.

We prospectively tested all blood donors between February and April 1998 for serum anti-HCV antibodies by both an HCV third-generation enzyme-linked immunosorbent assay (ELISA) (Abbott HCV EIA 3.0 test, Abbott, Chicago, IL, USA), which was chosen as a reference test for immunoglobulin (Ig) G antibodies to HCV, and by a simple membrane immunoassay system (Ortho HCV Ab Quik Pack, Ortho Diagnostic Systems Inc., Tokyo, Japan) (7). Anti-HCV-positive serum samples were further subjected to qualitative detection of HCV RNA by reverse transcription-polymerase chain reaction (AMPLICOR-HCV, Roche Diagnostic Systems, Inc., Branchburg, NJ, USA) (8). Of 163 serum samples (mean age ± standard deviation, 30±8 years), 155 were from male blood donors, 83 (51%) from first-time donors, and 125 (77%) from donors in the recipient's family. Fifteen (9.2%; 95% confidence interval [CI] 5%-15%) samples contained IgG to HCV by ELISA. Of the ELISA-positive samples, 14 were positive by the Quik Pack assay (sensitivity, 93.0%); of the 148 remaining ELISA-negative samples, 147 were negative by the Quik Pack assay (specificity, 99.3%). The agreement between the results of the two methods was 98.7%. Of the 163 samples, 10 (6.1%; CI 95%: 3%-11%) were positive for HCV antibodies (by ELISA and rapid test) and for HCV RNA.

We confirmed a high prevalence of HCV-seropositivity among blood donors in Bangui and the subsequent high rate of HCV RNA viremic blood donations. To offset the major risk for transfusion-acquired HCV in Central Africa we recommend screening donated blood for anti-HCV. When laboratory facilities to perform ELISA are not available, the Quik Pack system, a simple reliable method for detecting anti-HCV antibodies in human serum that requires neither complex reagent preparation nor expensive instrumentation, could prove useful.

**Nicole Cancré,* Gérard Grésenguet,† François-Xavier Mbopi-Kéou, ‡Alain Kozemaka,† Ali Si Mohamed,* Mathieu Matta,* Jean-Jacques Fournel,§ and Laurent Bélec***
*Université Pierre et Marie Curie, Hôpital Broussais, Paris, France; †Centre National de Transfusion Sanguine, Bangui, République Centrafricaine; ‡London School of Hygiene and Tropical Medicine, London, United-Kingdom; and §Hôpital de la Pitié-Salpêtrière, Paris, France

### References

1. Ndumbe PM, Skalsky J. Hepatitis C virus infection in different populations in Cameroon. Scand J Infect Dis 1993;25:689-92.
2. Xu LZ, Larzul D, Delaporte E, Bréchot C, Kremsdorf D. Hepatitis C virus genotype 4 highly prevalent in Central Africa (Gabon). J Gen Virol 1994;75:2393-8.
3. Fretz C, Jeannel D, Stuyver L, Herve V, Lunel F, Boudifa A, et al. HCV Infection in a rural population of Central African Republic (CAR): evidence for three additional subtypes of genotype 4. J Med Virol 1995;47:435-7.
4. Pawlotsky JM, Bélec L, Grésenguet G, Desforges L, Bouvier M, Duval J, et al. High prevalence of hepatitis B, C and E markers in young sexually active adults from the Central African Republic. J Med Virol 1995;46:269-73.
5. Aceti A, Taliani D. Hepatitis C virus testing in African sera. Ann Intern Med 1992;116:427.
6. Callahan JD, Constantine NT, Kataaha P, Zhang X, Hyams KC, Bansal J. Second generation hepatitis C virus assays: performance when testing African sera. J Med Virol 1993;41:35-8.
7. Kodama T, Ichiyama S, Sato K, Nada T, Nakashima N. Evaluation of a membrane filter assay system, Ortho HCV Ab Quik Pack, for detection of anti-hepatitis C virus antibody. J Clin Microbiol 1998;36:1439-40.
8. Young KKY, R. Resnick RM, Myers TW. Detection of hepatitis C virus RNA by a combined reverse transcription-polymerase chain reaction assay. J Clin Microbiol 1993;31:882-6.

### Immunization of Peacekeeping Forces[1]

**To the Editor:** The immunization status of military contingents arriving from different nations for peacekeeping missions may vary widely. This variation results from lack of information, coordination, and financial support.

For larger missions, the United Nations (UN) Headquarters issues recommendations about needed vaccines; recently, operations officers have consulted World Health Organization experts before issuing recommendations, and their advice, which takes into account epidemiologic data in the host country, has improved. Medical officers who develop recommendations for smaller missions must consider the pathogenic agent; environment; host efficacy, safety, and price of preventive measures; and legal and ethical aspects.

Data on the incidence of vaccine-preventable diseases within a military population that had similar duties in the same location are rarely available. When data from the respective region are not available, disease incidence or prevalence in the host country may be substituted. These data, however, may be misleading since the military often does not have the same lifestyle as the native population. Plague, for instance, had an incidence rate of 8 per 100,000 in Namibia, but not a single case was reported in the South African Armed Forces (unpub. SAMS report: Disease Profile of South West Africa, 1989). If epidemiologic documentation for a host country is not available, data from neighboring countries may be useful.

Traveler's diarrhea is the most frequent health problem abroad (1,2). Although the diarrhea is self-limited and lasts an average of 1 day with appropriate treatment (4 days without), the unproductive time may be detrimental to a military mission. Oral vaccines against the three most frequent causes of traveler's diarrhea (enterotoxigenic *Escherichia coli*, *Campylobacter* spp., and rotavirus [1,2]) are being developed; the latter will be available soon (3). Hepatitis A, most frequent among the vaccine-preventable diseases (4), is 10 to 100 times more frequent than typhoid fever (4,5). Hepatitis B occurs mainly in expatriates, but infections have also been observed in tourists who have had unprotected casual sex (6). The incidence rate of rabies is unknown, but animal bites that may result in rabies virus transmission and thus necessitate postexposure prophylaxis are frequent (7). Only anecdotal cases of diphtheria, tetanus, and tuberculosis have been reported (8). Poliomyelitis, yellow fever, Japanese encephalitis, and plague occur only in limited parts of the world (5). The situation may rapidly change as

[1]Presented in part at the NATO Research & Technology Organization, Aerospace Medical Panel Symposium on Aeromedical Support Issues in Contingency Operations, Rotterdam, The Netherlands, 1 October 1997.

